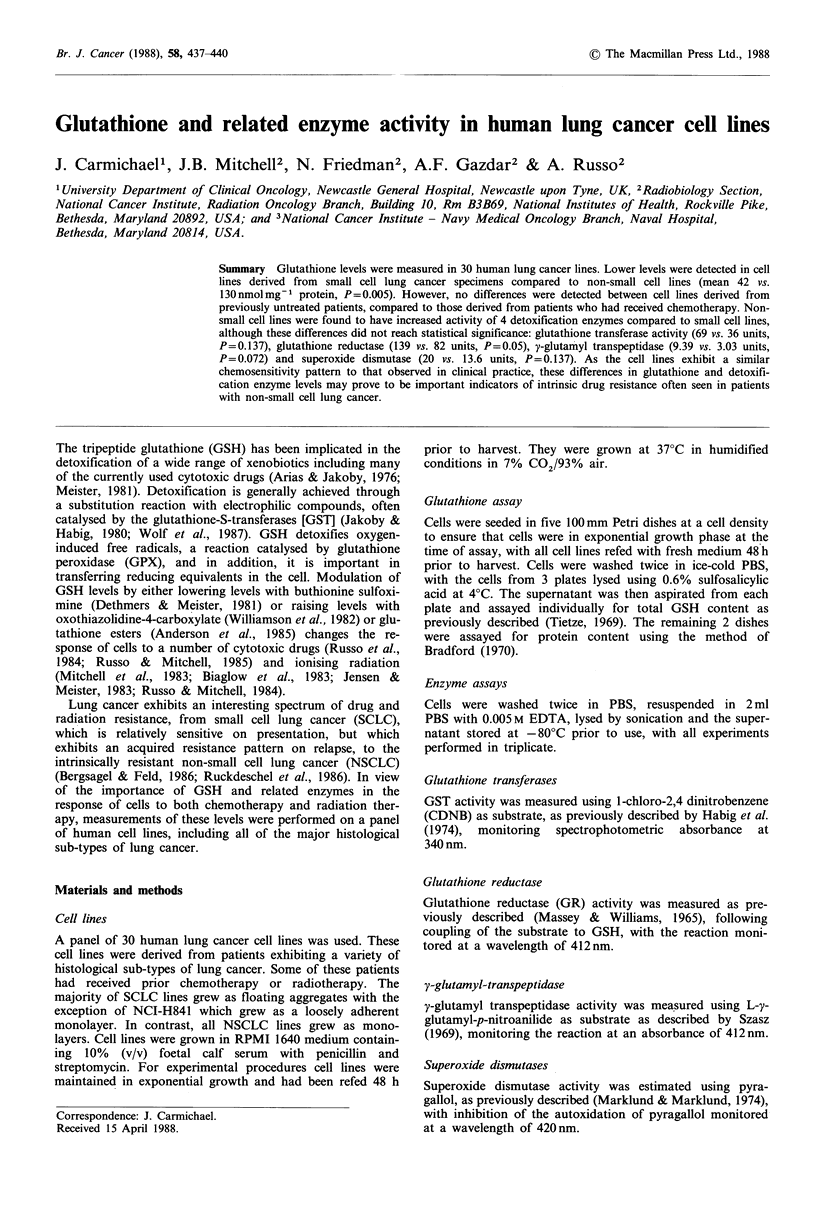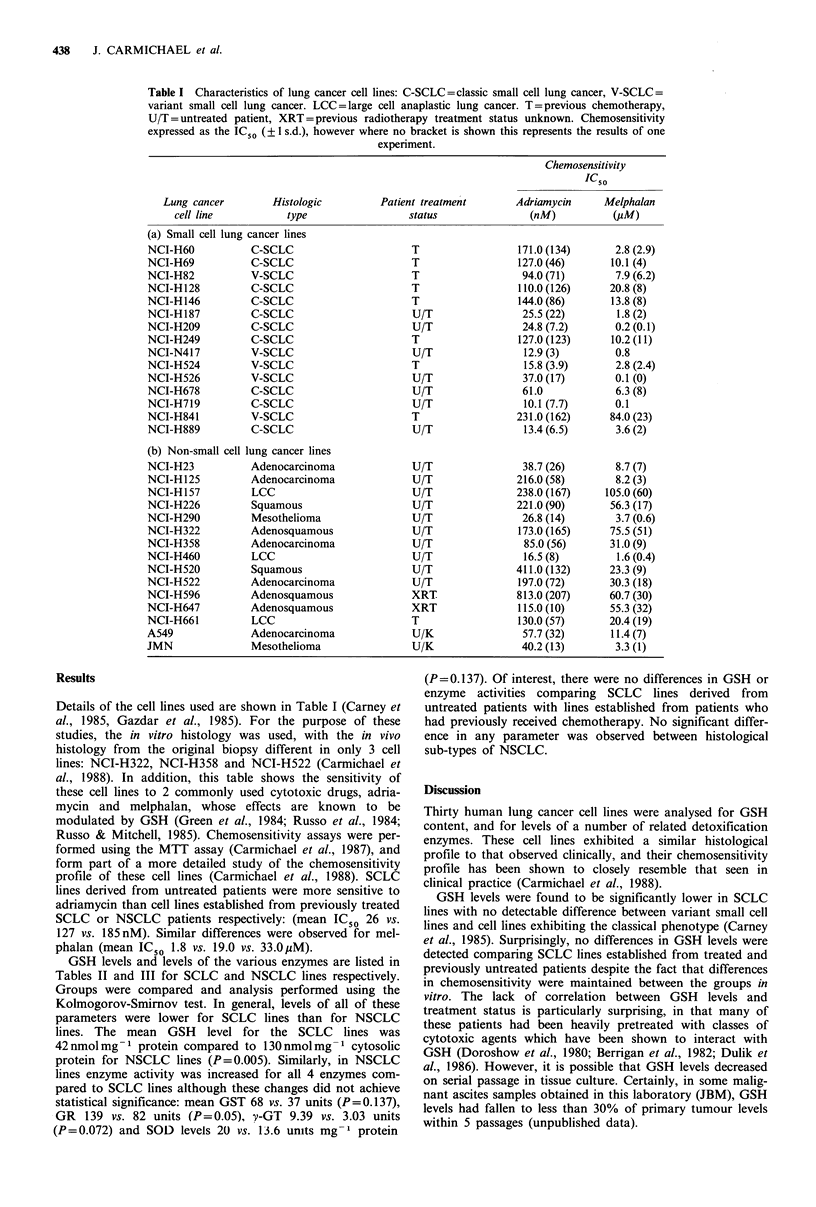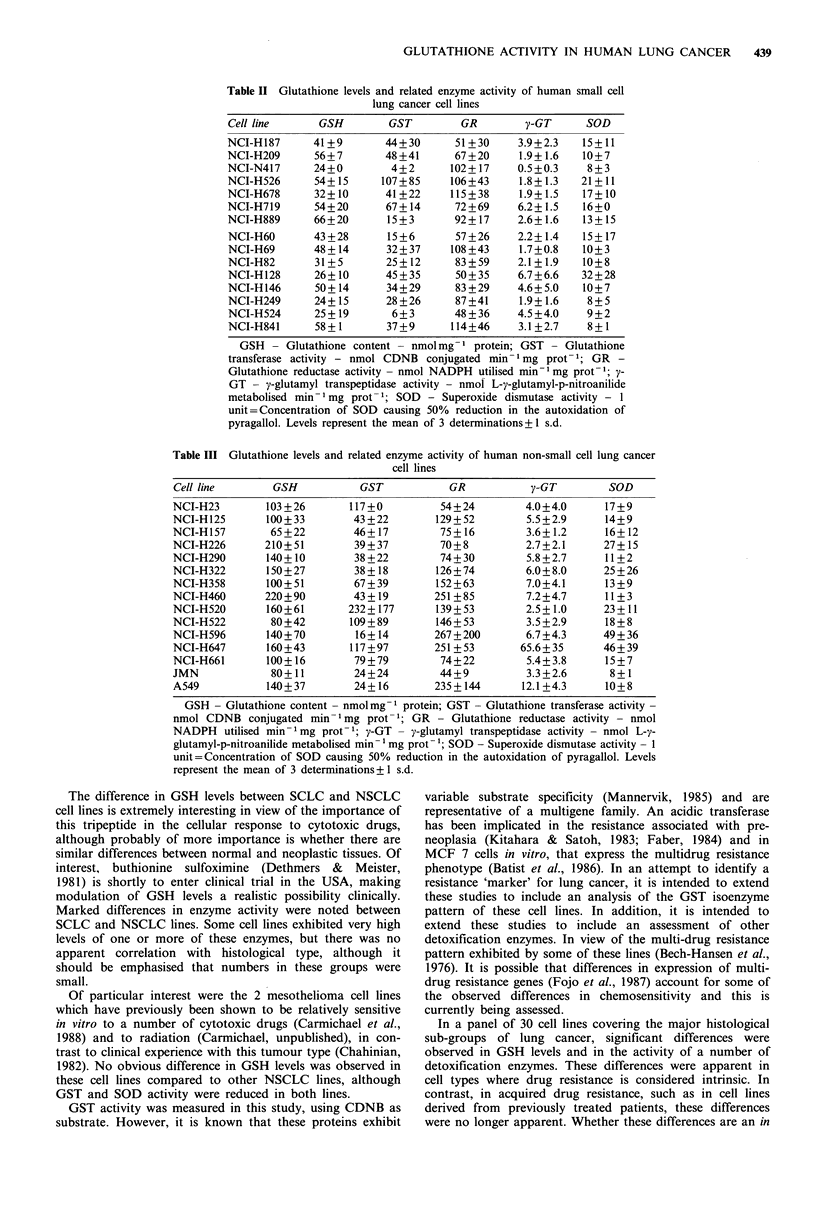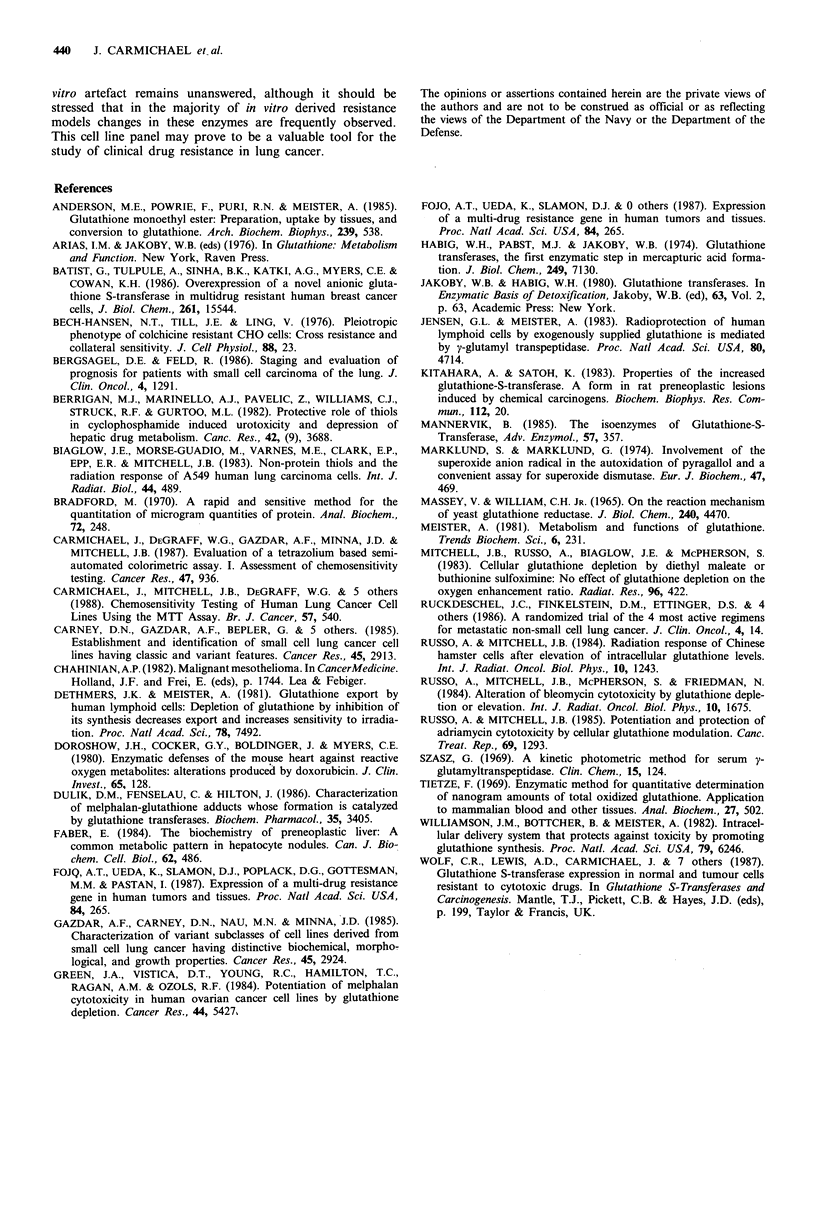# Glutathione and related enzyme activity in human lung cancer cell lines.

**DOI:** 10.1038/bjc.1988.236

**Published:** 1988-10

**Authors:** J. Carmichael, J. B. Mitchell, N. Friedman, A. F. Gazdar, A. Russo

**Affiliations:** University Department of Clinical Oncology, Newcastle General Hospital, Newcastle upon Tyne, UK.

## Abstract

Glutathione levels were measured in 30 human lung cancer lines. Lower levels were detected in cell lines derived from small cell lung cancer specimens compared to non-small cell lines (mean 42 vs. 130 nmol mg-1 protein, P = 0.005). However, no difference were detected between cell lines derived from previously untreated patients, compared to those derived from patients who had received chemotherapy. Non-small cell lines were found to have increased activity of 4 detoxification enzymes compared to small cell lines, although these differences did not reach statistical significance: glutathione transferase activity (69 vs. 36 units, P = 0.137), glutathione reductase (139 vs. 82 units, P = 0.05), gamma-glutamyl transpeptidase (9.39 vs. 3.03 units, P = 0.072) and superoxide dismutase (20 vs. 13.6 units, P = 0.137). As the cell lines exhibit a similar chemosensitivity pattern to that observed in clinical practice, these differences in glutathione and detoxification enzyme levels may prove to be important indicators of intrinsic drug resistance often seen in patients with non-small cell lung cancer.


*Br. J. Cancer* (1988), **58**, 437–440 © The Macmillan Press Ltd., 1988

### Glutathione and related enzyme activity in human lung cancer cell lines

J. Carmichael[1], J.B. Mitchell[2], N. Friedman[2], A.F. Gazdar[2] & A. Russo[2]

[1]*University Department of Clinical Oncology, Newcastle General Hospital, Newcastle upon Tyne, UK,* [2]*Radiobiology Section, National Cancer Institute, Radiation Oncology Branch, Building 10, Rm B3B69, National Institutes of Health, Rockville Pike, Bethesda, Maryland 20892, USA; and* [3]*National Cancer Institute – Navy Medical Oncology Branch, Naval Hospital, Bethesda, Maryland 20814, USA.*

**Summary** Glutathione levels were measured in 30 human lung cancer lines. Lower levels were detected in cell lines derived from small cell lung cancer specimens compared to non-small cell lines (mean 42 *vs.* 130 nmol $mg^{-1}$ protein, $P=0.005$). However, no differences were detected between cell lines derived from previously untreated patients, compared to those derived from patients who had received chemotherapy. Non-small cell lines were found to have increased activity of 4 detoxification enzymes compared to small cell lines, although these differences did not reach statistical significance: glutathione transferase activity (69 *vs.* 36 units, $P=0.137$), glutathione reductase (139 *vs.* 82 units, $P=0.05$), γ-glutamyl transpeptidase (9.39 *vs.* 3.03 units, $P=0.072$) and superoxide dismutase (20 *vs.* 13.6 units, $P=0.137$). As the cell lines exhibit a similar chemosensitivity pattern to that observed in clinical practice, these differences in glutathione and detoxification enzyme levels may prove to be important indicators of intrinsic drug resistance often seen in patients with non-small cell lung cancer.

The tripeptide glutathione (GSH) has been implicated in the detoxification of a wide range of xenobiotics including many of the currently used cytotoxic drugs (Arias & Jakoby, 1976; Meister, 1981). Detoxification is generally achieved through a substitution reaction with electrophilic compounds, often catalysed by the glutathione-S-transferases [GST] (Jakoby & Habig, 1980; Wolf *et al.*, 1987). GSH detoxifies oxygen-induced free radicals, a reaction catalysed by glutathione peroxidase (GPX), and in addition, it is important in transferring reducing equivalents in the cell. Modulation of GSH levels by either lowering levels with buthionine sulfoximine (Dethmers & Meister, 1981) or raising levels with oxothiazolidine-4-carboxylate (Williamson *et al.*, 1982) or glutathione esters (Anderson *et al.*, 1985) changes the response of cells to a number of cytotoxic drugs (Russo *et al.*, 1984; Russo & Mitchell, 1985) and ionising radiation (Mitchell *et al.*, 1983; Biaglow *et al.*, 1983; Jensen & Meister, 1983; Russo & Mitchell, 1984).

Lung cancer exhibits an interesting spectrum of drug and radiation resistance, from small cell lung cancer (SCLC), which is relatively sensitive on presentation, but which exhibits an acquired resistance pattern on relapse, to the intrinsically resistant non-small cell lung cancer (NSCLC) (Bergsagel & Feld, 1986; Ruckdeschel *et al.*, 1986). In view of the importance of GSH and related enzymes in the response of cells to both chemotherapy and radiation therapy, measurements of these levels were performed on a panel of human cell lines, including all of the major histological sub-types of lung cancer.

### Materials and methods

### *Cell lines*

A panel of 30 human lung cancer cell lines was used. These cell lines were derived from patients exhibiting a variety of histological sub-types of lung cancer. Some of these patients had received prior chemotherapy or radiotherapy. The majority of SCLC lines grew as floating aggregates with the exception of NCI-H841 which grew as a loosely adherent monolayer. In contrast, all NSCLC lines grew as monolayers. Cell lines were grown in RPMI 1640 medium containing 10% (v/v) foetal calf serum with penicillin and streptomycin. For experimental procedures cell lines were maintained in exponential growth and had been refed 48 h prior to harvest. They were grown at 37°C in humidified conditions in 7% $CO_2$/93% air.

Correspondence: J. Carmichael.
Received 15 April 1988.

### *Glutathione assay*

Cells were seeded in five 100 mm Petri dishes at a cell density to ensure that cells were in exponential growth phase at the time of assay, with all cell lines refed with fresh medium 48 h prior to harvest. Cells were washed twice in ice-cold PBS, with the cells from 3 plates lysed using 0.6% sulfosalicylic acid at 4°C. The supernatant was then aspirated from each plate and assayed individually for total GSH content as previously described (Tietze, 1969). The remaining 2 dishes were assayed for protein content using the method of Bradford (1970).

### *Enzyme assays*

Cells were washed twice in PBS, resuspended in 2 ml PBS with 0.005 M EDTA, lysed by sonication and the supernatant stored at −80°C prior to use, with all experiments performed in triplicate.

### *Glutathione transferases*

GST activity was measured using 1-chloro-2,4 dinitrobenzene (CDNB) as substrate, as previously described by Habig *et al.* (1974), monitoring spectrophotometric absorbance at 340 nm.

### *Glutathione reductase*

Glutathione reductase (GR) activity was measured as previously described (Massey & Williams, 1965), following coupling of the substrate to GSH, with the reaction monitored at a wavelength of 412 nm.

### *γ-glutamyl-transpeptidase*

γ-glutamyl transpeptidase activity was measured using L-γ-glutamyl-*p*-nitroanilide as substrate as described by Szasz (1969), monitoring the reaction at an absorbance of 412 nm.

### *Superoxide dismutases*

Superoxide dismutase activity was estimated using pyragallol, as previously described (Marklund & Marklund, 1974), with inhibition of the autoxidation of pyragallol monitored at a wavelength of 420 nm.

Table I Characteristics of lung cancer cell lines: C-SCLC = classic small cell lung cancer, V-SCLC = variant small cell lung cancer. LCC = large cell anaplastic lung cancer. T = previous chemotherapy, U/T = untreated patient, XRT = previous radiotherapy treatment status unknown. Chemosensitivity expressed as the $IC_{50}$ ($\pm 1$ s.d.), however where no bracket is shown this represents the results of one experiment.

| Lung cancer cell line | Histologic type | Patient treatment status | Chemosensitivity $IC_{50}$ Adriamycin (nM) | Chemosensitivity $IC_{50}$ Melphalan (μM) |
|---|---|---|---|---|
| (a) Small cell lung cancer lines | | | | |
| NCI-H60 | C-SCLC | T | 171.0 (134) | 2.8 (2.9) |
| NCI-H69 | C-SCLC | T | 127.0 (46) | 10.1 (4) |
| NCI-H82 | V-SCLC | T | 94.0 (71) | 7.9 (6.2) |
| NCI-H128 | C-SCLC | T | 110.0 (126) | 20.8 (8) |
| NCI-H146 | C-SCLC | T | 144.0 (86) | 13.8 (8) |
| NCI-H187 | C-SCLC | U/T | 25.5 (22) | 1.8 (2) |
| NCI-H209 | C-SCLC | U/T | 24.8 (7.2) | 0.2 (0.1) |
| NCI-H249 | C-SCLC | T | 127.0 (123) | 10.2 (11) |
| NCI-N417 | V-SCLC | U/T | 12.9 (3) | 0.8 |
| NCI-H524 | V-SCLC | T | 15.8 (3.9) | 2.8 (2.4) |
| NCI-H526 | V-SCLC | U/T | 37.0 (17) | 0.1 (0) |
| NCI-H678 | C-SCLC | U/T | 61.0 | 6.3 (8) |
| NCI-H719 | C-SCLC | U/T | 10.1 (7.7) | 0.1 |
| NCI-H841 | V-SCLC | T | 231.0 (162) | 84.0 (23) |
| NCI-H889 | C-SCLC | U/T | 13.4 (6.5) | 3.6 (2) |
| (b) Non-small cell lung cancer lines | | | | |
| NCI-H23 | Adenocarcinoma | U/T | 38.7 (26) | 8.7 (7) |
| NCI-H125 | Adenocarcinoma | U/T | 216.0 (58) | 8.2 (3) |
| NCI-H157 | LCC | U/T | 238.0 (167) | 105.0 (60) |
| NCI-H226 | Squamous | U/T | 221.0 (90) | 56.3 (17) |
| NCI-H290 | Mesothelioma | U/T | 26.8 (14) | 3.7 (0.6) |
| NCI-H322 | Adenosquamous | U/T | 173.0 (165) | 75.5 (51) |
| NCI-H358 | Adenocarcinoma | U/T | 85.0 (56) | 31.0 (9) |
| NCI-H460 | LCC | U/T | 16.5 (8) | 1.6 (0.4) |
| NCI-H520 | Squamous | U/T | 411.0 (132) | 23.3 (9) |
| NCI-H522 | Adenocarcinoma | U/T | 197.0 (72) | 30.3 (18) |
| NCI-H596 | Adenosquamous | XRT | 813.0 (207) | 60.7 (30) |
| NCI-H647 | Adenosquamous | XRT | 115.0 (10) | 55.3 (32) |
| NCI-H661 | LCC | T | 130.0 (57) | 20.4 (19) |
| A549 | Adenocarcinoma | U/K | 57.7 (32) | 11.4 (7) |
| JMN | Mesothelioma | U/K | 40.2 (13) | 3.3 (1) |

### Results

Details of the cell lines used are shown in Table I (Carney *et al.*, 1985, Gazdar *et al.*, 1985). For the purpose of these studies, the *in vitro* histology was used, with the *in vivo* histology from the original biopsy different in only 3 cell lines: NCI-H322, NCI-H358 and NCI-H522 (Carmichael *et al.*, 1988). In addition, this table shows the sensitivity of these cell lines to 2 commonly used cytotoxic drugs, adriamycin and melphalan, whose effects are known to be modulated by GSH (Green *et al.*, 1984; Russo *et al.*, 1984; Russo & Mitchell, 1985). Chemosensitivity assays were performed using the MTT assay (Carmichael *et al.*, 1987), and form part of a more detailed study of the chemosensitivity profile of these cell lines (Carmichael *et al.*, 1988). SCLC lines derived from untreated patients were more sensitive to adriamycin than cell lines established from previously treated SCLC or NSCLC patients respectively: (mean $IC_{50}$ 26 *vs.* 127 *vs.* 185 nM). Similar differences were observed for melphalan (mean $IC_{50}$ 1.8 *vs.* 19.0 *vs.* 33.0 μM).

GSH levels and levels of the various enzymes are listed in Tables II and III for SCLC and NSCLC lines respectively. Groups were compared and analysis performed using the Kolmogorov-Smirnov test. In general, levels of all of these parameters were lower for SCLC lines than for NSCLC lines. The mean GSH level for the SCLC lines was 42 nmol $mg^{-1}$ protein compared to 130 nmol $mg^{-1}$ cytosolic protein for NSCLC lines ($P = 0.005$). Similarly, in NSCLC lines enzyme activity was increased for all 4 enzymes compared to SCLC lines although these changes did not achieve statistical significance: mean GST 68 *vs.* 37 units ($P = 0.137$), GR 139 *vs.* 82 units ($P = 0.05$), γ-GT 9.39 *vs.* 3.03 units ($P = 0.072$) and SOD levels 20 *vs.* 13.6 units $mg^{-1}$ protein ($P = 0.137$). Of interest, there were no differences in GSH or enzyme activities comparing SCLC lines derived from untreated patients with lines established from patients who had previously received chemotherapy. No significant difference in any parameter was observed between histological sub-types of NSCLC.

### Discussion

Thirty human lung cancer cell lines were analysed for GSH content, and for levels of a number of related detoxification enzymes. These cell lines exhibited a similar histological profile to that observed clinically, and their chemosensitivity profile has been shown to closely resemble that seen in clinical practice (Carmichael *et al.*, 1988).

GSH levels were found to be significantly lower in SCLC lines with no detectable difference between variant small cell lines and cell lines exhibiting the classical phenotype (Carney *et al.*, 1985). Surprisingly, no differences in GSH levels were detected comparing SCLC lines established from treated and previously untreated patients despite the fact that differences in chemosensitivity were maintained between the groups *in vitro*. The lack of correlation between GSH levels and treatment status is particularly surprising, in that many of these patients had been heavily pretreated with classes of cytotoxic agents which have been shown to interact with GSH (Doroshow *et al.*, 1980; Berrigan *et al.*, 1982; Dulik *et al.*, 1986). However, it is possible that GSH levels decreased on serial passage in tissue culture. Certainly, in some malignant ascites samples obtained in this laboratory (JBM), GSH levels had fallen to less than 30% of primary tumour levels within 5 passages (unpublished data).

**Table II** Glutathione levels and related enzyme activity of human small cell lung cancer cell lines

| *Cell line* | *GSH* | *GST* | *GR* | *γ-GT* | *SOD* |
|---|---|---|---|---|---|
| NCI-H187 | 41±9 | 44±30 | 51±30 | 3.9±2.3 | 15±11 |
| NCI-H209 | 56±7 | 48±41 | 67±20 | 1.9±1.6 | 10±7 |
| NCI-N417 | 24±0 | 4±2 | 102±17 | 0.5±0.3 | 8±3 |
| NCI-H526 | 54±15 | 107±85 | 106±43 | 1.8±1.3 | 21±11 |
| NCI-H678 | 32±10 | 41±22 | 115±38 | 1.9±1.5 | 17±10 |
| NCI-H719 | 54±20 | 67±14 | 72±69 | 6.2±1.5 | 16±0 |
| NCI-H889 | 66±20 | 15±3 | 92±17 | 2.6±1.6 | 13±15 |
| NCI-H60 | 43±28 | 15±6 | 57±26 | 2.2±1.4 | 15±17 |
| NCI-H69 | 48±14 | 32±37 | 108±43 | 1.7±0.8 | 10±3 |
| NCI-H82 | 31±5 | 25±12 | 83±59 | 2.1±1.9 | 10±8 |
| NCI-H128 | 26±10 | 45±35 | 50±35 | 6.7±6.6 | 32±28 |
| NCI-H146 | 50±14 | 34±29 | 83±29 | 4.6±5.0 | 10±7 |
| NCI-H249 | 24±15 | 28±26 | 87±41 | 1.9±1.6 | 8±5 |
| NCI-H524 | 25±19 | 6±3 | 48±36 | 4.5±4.0 | 9±2 |
| NCI-H841 | 58±1 | 37±9 | 114±46 | 3.1±2.7 | 8±1 |

GSH – Glutathione content – nmol mg$^{-1}$ protein; GST – Glutathione transferase activity – nmol CDNB conjugated min$^{-1}$ mg prot$^{-1}$; GR – Glutathione reductase activity – nmol NADPH utilised min$^{-1}$ mg prot$^{-1}$; γ-GT – γ-glutamyl transpeptidase activity – nmol L-γ-glutamyl-p-nitroanilide metabolised min$^{-1}$ mg prot$^{-1}$; SOD – Superoxide dismutase activity – 1 unit = Concentration of SOD causing 50% reduction in the autoxidation of pyragallol. Levels represent the mean of 3 determinations ± 1 s.d.

**Table III** Glutathione levels and related enzyme activity of human non-small cell lung cancer cell lines

| *Cell line* | *GSH* | *GST* | *GR* | *γ-GT* | *SOD* |
|---|---|---|---|---|---|
| NCI-H23 | 103±26 | 117±0 | 54±24 | 4.0±4.0 | 17±9 |
| NCI-H125 | 100±33 | 43±22 | 129±52 | 5.5±2.9 | 14±9 |
| NCI-H157 | 65±22 | 46±17 | 75±16 | 3.6±1.2 | 16±12 |
| NCI-H226 | 210±51 | 39±37 | 70±8 | 2.7±2.1 | 27±15 |
| NCI-H290 | 140±10 | 38±22 | 74±30 | 5.8±2.7 | 11±2 |
| NCI-H322 | 150±27 | 38±18 | 126±74 | 6.0±8.0 | 25±26 |
| NCI-H358 | 100±51 | 67±39 | 152±63 | 7.0±4.1 | 13±9 |
| NCI-H460 | 220±90 | 43±19 | 251±85 | 7.2±4.7 | 11±3 |
| NCI-H520 | 160±61 | 232±177 | 139±53 | 2.5±1.0 | 23±11 |
| NCI-H522 | 80±42 | 109±89 | 146±53 | 3.5±2.9 | 18±8 |
| NCI-H596 | 140±70 | 16±14 | 267±200 | 6.7±4.3 | 49±36 |
| NCI-H647 | 160±43 | 117±97 | 251±53 | 65.6±35 | 46±39 |
| NCI-H661 | 100±16 | 79±79 | 74±22 | 5.4±3.8 | 15±7 |
| JMN | 80±11 | 24±24 | 44±9 | 3.3±2.6 | 8±1 |
| A549 | 140±37 | 24±16 | 235±144 | 12.1±4.3 | 10±8 |

GSH – Glutathione content – nmol mg$^{-1}$ protein; GST – Glutathione transferase activity – nmol CDNB conjugated min$^{-1}$ mg prot$^{-1}$; GR – Glutathione reductase activity – nmol NADPH utilised min$^{-1}$ mg prot$^{-1}$; γ-GT – γ-glutamyl transpeptidase activity – nmol L-γ-glutamyl-p-nitroanilide metabolised min$^{-1}$ mg prot$^{-1}$; SOD – Superoxide dismutase activity – 1 unit = Concentration of SOD causing 50% reduction in the autoxidation of pyragallol. Levels represent the mean of 3 determinations ± 1 s.d.

The difference in GSH levels between SCLC and NSCLC cell lines is extremely interesting in view of the importance of this tripeptide in the cellular response to cytotoxic drugs, although probably of more importance is whether there are similar differences between normal and neoplastic tissues. Of interest, buthionine sulfoximine (Dethmers & Meister, 1981) is shortly to enter clinical trial in the USA, making modulation of GSH levels a realistic possibility clinically. Marked differences in enzyme activity were noted between SCLC and NSCLC lines. Some cell lines exhibited very high levels of one or more of these enzymes, but there was no apparent correlation with histological type, although it should be emphasised that numbers in these groups were small.

Of particular interest were the 2 mesothelioma cell lines which have previously been shown to be relatively sensitive *in vitro* to a number of cytotoxic drugs (Carmichael *et al.*, 1988) and to radiation (Carmichael, unpublished), in contrast to clinical experience with this tumour type (Chahinian, 1982). No obvious difference in GSH levels was observed in these cell lines compared to other NSCLC lines, although GST and SOD activity were reduced in both lines.

GST activity was measured in this study, using CDNB as substrate. However, it is known that these proteins exhibit variable substrate specificity (Mannervik, 1985) and are representative of a multigene family. An acidic transferase has been implicated in the resistance associated with preneoplasia (Kitahara & Satoh, 1983; Faber, 1984) and in MCF 7 cells *in vitro*, that express the multidrug resistance phenotype (Batist *et al.*, 1986). In an attempt to identify a resistance 'marker' for lung cancer, it is intended to extend these studies to include an analysis of the GST isoenzyme pattern of these cell lines. In addition, it is intended to extend these studies to include an assessment of other detoxification enzymes. In view of the multi-drug resistance pattern exhibited by some of these lines (Bech-Hansen *et al.*, 1976). It is possible that differences in expression of multidrug resistance genes (Fojo *et al.*, 1987) account for some of the observed differences in chemosensitivity and this is currently being assessed.

In a panel of 30 cell lines covering the major histological sub-groups of lung cancer, significant differences were observed in GSH levels and in the activity of a number of detoxification enzymes. These differences were apparent in cell types where drug resistance is considered intrinsic. In contrast, in acquired drug resistance, such as in cell lines derived from previously treated patients, these differences were no longer apparent. Whether these differences are an *in*

vitro artefact remains unanswered, although it should be stressed that in the majority of *in vitro* derived resistance models changes in these enzymes are frequently observed. This cell line panel may prove to be a valuable tool for the study of clinical drug resistance in lung cancer.

The opinions or assertions contained herein are the private views of the authors and are not to be construed as official or as reflecting the views of the Department of the Navy or the Department of the Defense.

### References

ANDERSON, M.E., POWRIE, F., PURI, R.N. & MEISTER, A. (1985). Glutathione monoethyl ester: Preparation, uptake by tissues, and conversion to glutathione. *Arch. Biochem. Biophys.*, **239**, 538.

ARIAS, I.M. & JAKOBY, W.B. (eds) (1976). In *Glutathione: Metabolism and Function*. New York, Raven Press.

BATIST, G., TULPULE, A., SINHA, B.K., KATKI, A.G., MYERS, C.E. & COWAN, K.H. (1986). Overexpression of a novel anionic glutathione S-transferase in multidrug resistant human breast cancer cells, *J. Biol. Chem.*, **261**, 15544.

BECH-HANSEN, N.T., TILL, J.E. & LING, V. (1976). Pleiotropic phenotype of colchicine resistant CHO cells: Cross resistance and collateral sensitivity. *J. Cell Physiol.*, **88**, 23.

BERGSAGEL, D.E. & FELD, R. (1986). Staging and evaluation of prognosis for patients with small cell carcinoma of the lung. *J. Clin. Oncol.*, **4**, 1291.

BERRIGAN, M.J., MARINELLO, A.J., PAVELIC, Z., WILLIAMS, C.J., STRUCK, R.F. & GURTOO, M.L. (1982). Protective role of thiols in cyclophosphamide induced urotoxicity and depression of hepatic drug metabolism. *Canc. Res.*, **42**, (9), 3688.

BIAGLOW, J.E., MORSE-GUADIO, M., VARNES, M.E., CLARK, E.P., EPP, E.R. & MITCHELL, J.B. (1983). Non-protein thiols and the radiation response of A549 human lung carcinoma cells. *Int. J. Radiat. Biol.*, **44**, 489.

BRADFORD, M. (1970). A rapid and sensitive method for the quantitation of microgram quantities of protein. *Anal. Biochem.*, **72**, 248.

CARMICHAEL, J., DEGRAFF, W.G., GAZDAR, A.F., MINNA, J.D. & MITCHELL, J.B. (1987). Evaluation of a tetrazolium based semi-automated colorimetric assay. I. Assessment of chemosensitivity testing. *Cancer Res.*, **47**, 936.

CARMICHAEL, J., MITCHELL, J.B., DEGRAFF, W.G. & 5 others (1988). Chemosensitivity Testing of Human Lung Cancer Cell Lines Using the MTT Assay. *Br. J. Cancer*, **57**, 540.

CARNEY, D.N., GAZDAR, A.F., BEPLER, G. & 5 others. (1985). Establishment and identification of small cell lung cancer cell lines having classic and variant features. *Cancer Res.*, **45**, 2913.

CHAHINIAN, A.P. (1982). Malignant mesothelioma. In *CancerMedicine*. Holland, J.F. and Frei, E. (eds), p. 1744. Lea & Febiger.

DETHMERS, J.K. & MEISTER, A. (1981). Glutathione export by human lymphoid cells: Depletion of glutathione by inhibition of its synthesis decreases export and increases sensitivity to irradiation. *Proc. Natl Acad. Sci.*, **78**, 7492.

DOROSHOW, J.H., COCKER, G.Y., BOLDINGER, J. & MYERS, C.E. (1980). Enzymatic defenses of the mouse heart against reactive oxygen metabolites: alterations produced by doxorubicin. *J. Clin. Invest.*, **65**, 128.

DULIK, D.M., FENSELAU, C. & HILTON, J. (1986). Characterization of melphalan-glutathione adducts whose formation is catalyzed by glutathione transferases. *Biochem. Pharmacol.*, **35**, 3405.

FABER, E. (1984). The biochemistry of preneoplastic liver: A common metabolic pattern in hepatocyte nodules. *Can. J. Biochem. Cell. Biol.*, **62**, 486.

FOJQ, A.T., UEDA, K., SLAMON, D.J., POPLACK, D.G., GOTTESMAN, M.M. & PASTAN, I. (1987). Expression of a multi-drug resistance gene in human tumors and tissues. *Proc. Natl Acad. Sci. USA*, **84**, 265.

GAZDAR, A.F., CARNEY, D.N., NAU, M.N. & MINNA, J.D. (1985). Characterization of variant subclasses of cell lines derived from small cell lung cancer having distinctive biochemical, morphological, and growth properties. *Cancer Res.*, **45**, 2924.

GREEN, J.A., VISTICA, D.T., YOUNG, R.C., HAMILTON, T.C., RAGAN, A.M. & OZOLS, R.F. (1984). Potentiation of melphalan cytotoxicity in human ovarian cancer cell lines by glutathione depletion. *Cancer Res.*, **44**, 5427.

FOJO, A.T., UEDA, K., SLAMON, D.J. & 0 others (1987). Expression of a multi-drug resistance gene in human tumors and tissues. *Proc. Natl Acad. Sci. USA*, **84**, 265.

HABIG, W.H., PABST, M.J. & JAKOBY, W.B. (1974). Glutathione transferases, the first enzymatic step in mercapturic acid formation. *J. Biol. Chem.*, **249**, 7130.

JAKOBY, W.B. & HABIG, W.H. (1980). Glutathione transferases. In *Enzymatic Basis of Detoxification*, Jakoby, W.B. (ed), **63**, Vol. 2, p. 63, Academic Press: New York.

JENSEN, G.L. & MEISTER, A. (1983). Radioprotection of human lymphoid cells by exogenously supplied glutathione is mediated by γ-glutamyl transpeptidase. *Proc. Natl Acad. Sci. USA*, **80**, 4714.

KITAHARA, A. & SATOH, K. (1983). Properties of the increased glutathione-S-transferase. A form in rat preneoplastic lesions induced by chemical carcinogens. *Biochem. Biophys. Res. Commun.*, **112**, 20.

MANNERVIK, B. (1985). The isoenzymes of Glutathione-S-Transferase, *Adv. Enzymol.*, **57**, 357.

MARKLUND, S. & MARKLUND, G. (1974). Involvement of the superoxide anion radical in the autoxidation of pyragallol and a convenient assay for superoxide dismutase. *Eur. J. Biochem.*, **47**, 469.

MASSEY, V. & WILLIAM, C.H. JR. (1965). On the reaction mechanism of yeast glutathione reductase. *J. Biol. Chem.*, **240**, 4470.

MEISTER, A. (1981). Metabolism and functions of glutathione. *Trends Biochem. Sci.*, **6**, 231.

MITCHELL, J.B., RUSSO, A., BIAGLOW, J.E. & MCPHERSON, S. (1983). Cellular glutathione depletion by diethyl maleate or buthionine sulfoximine: No effect of glutathione depletion on the oxygen enhancement ratio. *Radiat. Res.*, **96**, 422.

RUCKDESCHEL, J.C., FINKELSTEIN, D.M., ETTINGER, D.S. & 4 others (1986). A randomized trial of the 4 most active regimens for metastatic non-small cell lung cancer. *J. Clin. Oncol.*, **4**, 14.

RUSSO, A. & MITCHELL, J.B. (1984). Radiation response of Chinese hamster cells after elevation of intracellular glutathione levels. *Int. J. Radiat. Oncol. Biol. Phys.*, **10**, 1243.

RUSSO, A., MITCHELL, J.B., MCPHERSON, S. & FRIEDMAN, N. (1984). Alteration of bleomycin cytotoxicity by glutathione depletion or elevation. *Int. J. Radiat. Oncol. Biol. Phys.*, **10**, 1675.

RUSSO, A. & MITCHELL, J.B. (1985). Potentiation and protection of adriamycin cytotoxicity by cellular glutathione modulation. *Canc. Treat. Rep.*, **69**, 1293.

SZASZ, G. (1969). A kinetic photometric method for serum γ-glutamyltranspeptidase. *Clin. Chem.*, **15**, 124.

TIETZE, F. (1969). Enzymatic method for quantitative determination of nanogram amounts of total oxidized glutathione. Application to mammalian blood and other tissues. *Anal. Biochem.*, **27**, 502.

WILLIAMSON, J.M., BOTTCHER, B. & MEISTER, A. (1982). Intracellular delivery system that protects against toxicity by promoting glutathione synthesis. *Proc. Natl. Acad. Sci. USA*, **79**, 6246.

WOLF, C.R., LEWIS, A.D., CARMICHAEL, J. & 7 others (1987). Glutathione S-transferase expression in normal and tumour cells resistant to cytotoxic drugs. In *Glutathione S-Transferases and Carcinogenesis*. Mantle, T.J., Pickett, C.B. & Hayes, J.D. (eds), p. 199, Taylor & Francis, UK.